# Adrenal hyperplasias in childhood: An update

**DOI:** 10.3389/fendo.2022.937793

**Published:** 2022-08-03

**Authors:** Georgia Pitsava, Constantine A. Stratakis

**Affiliations:** ^1^ Division of Intramural Research, Division of Population Health Research, Eunice Kennedy Shriver National Institutes of Child Health and Human Development, National Institutes of Health, Bethesda, MD, United States; ^2^ Section on Endocrinology and Genetics, Eunice Kennedy Shriver National Institute of Child Health and Human Development, National Institutes of Health, Bethesda, MD, United States; ^3^ Human Genetics and Precision Medicine, Institute of Molecular Biology and Biotechnology of the Foundation for Research and Technology Hellas (IMBB-FORTH), Heraklion, Greece; ^4^ ELPEN Research Institute, ELPEN, Athens, Greece

**Keywords:** adrenal cortex, Cushing syndrome, adrenal hyperplasia, congenital adrenal hyperplasia, childhood tumors

## Abstract

Pediatric adrenocortical hyperplasias are rare; they usually present with Cushing syndrome (CS); of them, isolated micronodular adrenal disease and its variant, primary pigmented adrenocortical disease are the most commonly encountered. Most cases are due to defects in the cyclic AMP/protein kinase A (cAMP/PKA) pathway, although a few cases remain without an identified genetic defect. Another cause of adrenal hyperplasia in childhood is congenital adrenal hyperplasia, a group of autosomal recessive disorders that affect steroidogenic enzymes in the adrenal cortex. Clinical presentation varies and depends on the extent of the underlying enzymatic defect. The most common form is due to 21-hydroxylase deficiency; it accounts for more than 90% of the cases. In this article, we discuss the genetic etiology of adrenal hyperplasias in childhood.

## Introduction

Adrenocortical tumors (ACTs) are rare in childhood and adolescence (1). The majority of them (approximately 90%) are functional and thus they can present with hirsutism, acne, rapid clitoral or penile enlargement, sexual hair growth as well as increased height velocity (2). Usually, single tumors are benign unilateral adenomas but they can be malignant in rare cases and physicians should be suspicious of that as an early diagnosis impacts overall survival. Less often, patients can present with benign multinodular hyperplastic lesions including primary pigmented nodular adrenocortical disease (PPNAD), non-pigmented isolated micronodular adrenal disease (iMAD), and corticotropin (ACTH)-independent macronodular adrenal hyperplasia (AIMH) (3). These diseases usually present with cortisol excess or Cushing syndrome (CS). On the other hand, deficits of the steroidogenic enzymes of the adrenal cortex, also cause adrenal hyperplasia but they are usually associated with decreased cortisol synthesis. These diseases are known collectively as “congenital adrenal hyperplasia” or CAH, are all autosomal recessive and have a variable clinical presentation. We review in this report both types of adrenal hyperplasias and any updates on their molecular genetics.

## Adrenal gland: Embryology and early development

The human adrenal gland arises from two different embryological tissues: the cortex develops from the intermediate mesoderm of the urogenital ridge, while the medulla develops from the neural crest-derived chromaffin cells (4). The earliest recognizable form of the adrenal gland is called ‘adrenal primordium’ and appears at 28-30 days post conception (dpc); it is marked by the expression of steroidogenic factor-1 (SF1; also known as Ad4BP or NR5A1) which is essential for the adrenal development and steroidogenesis (5, 6). By the 7^th^-8^th^ week of fetal development, the developing adrenal cortex delineates two distinct components, the inner fetal zone (FZ) -that comprises 80-90% of the cortical volume- and the outer definitive zone (DZ) (5, 7). By the 9^th^ week of gestation, the developing adrenal gland becomes completely encapsulated (8). The FZ cells express cytochrome P450 17α (CYP17A1), an enzyme that has both 17 hydroxylase and 17,20 lyase activity and converts pregnenolone to dehydroepiandrosterone (DHEA). At this stage, DHEA is the main product produced at ZF; later, it undergoes conversion to estrogens by the placenta for the maintenance of normal pregnancy.

By the end of the second trimester, the transitional zone (between FZ and DZ) expresses *HSD3B2*, and thus cortisol synthesis is initiated in the fetus (9). By late gestation, the DZ has begun differentiating into the zona glomerulosa (ZG) and zona fasciculata (ZF). Soon after birth, the FZ undergoes regression due to increased apoptotic activity, resulting in decrease in the weight of the adrenal glands by approximately 50% (2, 10). Zona reticularis (ZR) begins to form later in childhood; around the age of 6-8 years in females and 7-9 years in males. This process is known as adrenarche and is characterized by the production of adrenal androgens (11). Medulla is not recognized as a distinct structure with the exception of small clusters of chromaffin cells scattered throughout the cortex, until after birth (12).

## Hypothalamic-pituitary-adrenal axis: Glucocorticoid secretion

Glucocorticoid secretion is regulated by the HPA axis. The most important components of the HPA axis include: 1) the corticotropin-releasing hormone (CRH)- whose actions are mediated by two receptors, CRHR1 and CRHR2- which is released, along with vasopressin, from the paraventricular nucleus of the hypothalamus and it is carried to the anterior pituitary where it stimulates 2) adrenocorticotropic hormone (ACTH) secreted from the anterior lobe of the pituitary gland, and 3) cortisol that is produced by the adrenal cortex in response to the binding of ACTH to the melanocortin type-2 receptor (MC2-R) in the ZF. The HPA axis is subject to the negative feedback cycle from the glucocorticoids; circulating cortisol inhibits primarily CRH and secondarily ACTH (13).

## Renin-angiotensin-aldosterone system: Mineralocorticoid secretion

RAAS is a vital regulator of systemic vascular resistance and blood pressure, by increasing vascular tone, water and sodium reabsorption. It has three main components that include renin, angiotensin II and aldosterone. Renin, a protease synthesized in the juxtaglomerular cells of the kidney, cleaves angiotensinogen -synthesized in the liver- to the biologically inactive angiotensin I (AngI). Following that, AngI is converted to AngII by the angiotensin converting enzyme (ACE), which is found primarily in the vascular endothelium of the lungs and kidneys. AngII acts by activating AngII type 1 (AT1) receptors in the adrenal cortex, kidneys and arterioles, resulting in aldosterone release, vasoconstriction, and blood pressure increase. Release of AngII -the major stimuli of RAAS activation- is triggered by intravascular volume depletion and hyperkalemia.

## Adrenal androgen secretion

The ZR in the adrenal glands produces the precursor steroids dehydroepiandrosterone (DHEA) and DHEA sulfate (DHEAS) which are later converted, in the peripheral tissues, to potent androgens and estrogens (14, 15). Their secretion is partially regulated by ACTH; however, in multiple instances, the exact mechanism has not been elucidated yet.

### Adrenal hyperplasias

Benign adrenocortical tumors producing cortisol.

#### Etiology

The vast majority of cortisol-producing adrenal hyperplasias and tumors harbor defects in the cAMP/PKA signaling pathway (16). Under normal circumstances, in the adrenocortical cells, ACTH binds to MC2R (a G protein-coupled receptor) that causes an increase of the cAMP levels and thus activation of the PKA. PKA phosphorylates various transcription factors including cAMP response element binding protein (CREB), steroidogenic factor 1 (SF-1) and activating transcription factor 1 (ATF-1). Following that, these transcription factors bind to the cAMP response element (CRE) in the nucleus and regulate the expression of genes important in steroidogenesis (17–20).

### Primary bilateral adrenal hyperplasias

This group of disorders can be categorized into two groups based on the size of the associated nodules: macronodular hyperplasias (BMAH, nodule size ≥1cm), which more commonly affect older adults and micronodular hyperplasias (MiBAH, nodule size <1cm) that occur more frequently in children and young adults. The latter includes primary pigmented adrenocortical nodular disease (PPNAD) and isolated micronodular adrenal disease (iMAD) (21). Two additional characteristics used for the classification of BAHs include the pigment that is present and whether there is atrophy or hyperplasia of the surrounding cortex tissue (22). The pigment most of the times is lipofuscin and macroscopically is seen as light to dark brown or, rarely, black (22).

### Primary pigmented adrenocortical nodular disease

PPNAD in children and adolescents has been linked to periodic or atypical CS (23–25). In PPNAD the adrenal glands appear to have multiple cortisol-secreting pigmented nodules usually <4-6mm in diameter. PPNAD can present as sporadic or familial; most commonly it presents as familial PPNAD as part of Carney complex (CNC, OMIM#160980). CNC, that was first described in 1985 (26), is a tumor predisposition syndrome that presents with multiple endocrine tumors in addition to lentigines and myxomas (21, 27). In 2000, germline inactivating variants in the *PRKAR1A* gene which encodes for the regulatory type 1α-subunit of PKA, mapped on the 17q22-24 chromosomal region, was identified as the causative gene in the majority of CNC cases (28–30). These variants lead to increased activity of the PKA. More genetic alterations in the cAMP/PKA signaling pathway have been identified over the last two decades in patients with MiBAH (31–33).

### Cushing syndrome

Cushing syndrome (CS) can be exogenous (or ‘iatrogenic’) when glucocorticoids are used in high doses for a prolonged period of time or endogenous due to overproduction of cortisol either by an ACTH-producing pituitary tumor (also known as Cushing disease -CD-) or by an ACTH-independent ACT. Endogenous CS has an incidence of 0.7-2.4 per million people per year (34); in children BAHs as etiologies of CS are much more common than in adults (23).

#### Clinical presentation

In most cases, CS onset is slow and gradual (35). The hallmark of CS in childhood is increasing weight with growth velocity deceleration. Other common presenting symptoms include headaches, facial plethora, hypertension, amenorrhea, hypogonadism and hirsutism (**Table 1**) (35–39). Virilization is not often present except for cases that the tumor produces androgens. Additional manifestations from skin such as abdominal striae, acne and bruising may also occur often.

**Table 1:** Phenotypic manifestations of pediatric Cushing syndrome.

Organ system	Manifestations
**Cardiovascular**	Hypertension, coagulopathy
**Gonadal**	Amenorrhea, virilization, gynecomastia
**Skin**	Acne, acanthosis nigricans, easy bruising, hirsutism, fine hair, striae
**CNS**	Anxiety, mood swings, depression, fatigue
**Other**	Facial plethora, supra-temporal and supra-clavicular fat pads, bone fractures, impaired glucose tolerance, nephrolithiasis

#### Diagnosis

**Figure 1:**
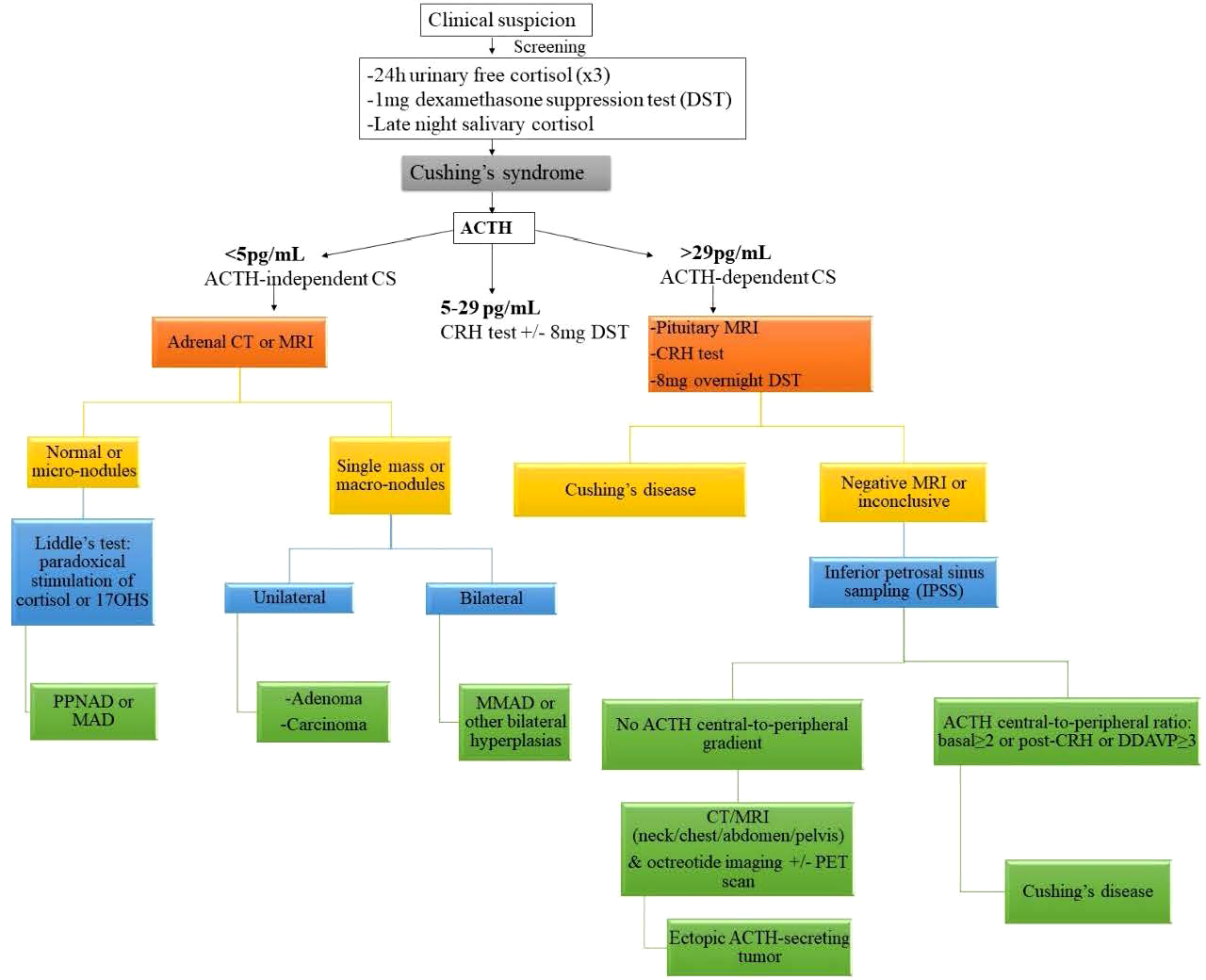
Diagnostic algorithm for suspected Cushing syndrome. ACTH adrenocorticotropic hormone, CS Cushing syndrome, CT computed tomography, DDAVP desmopressin, MAD micronodular adrenal disease, MRI magnetic resonance imaging, PET positron emission tomography, 17OHS 17-hydroxysteroid. Reprinted from Constantine A. Stratakis. Cushing Syndrome in Pediatrics. Endocrinology and Metabolism Clinics of North America. Volume 41, Issue 4, December 2012, Pages 793-803, with permission from Elsevier.

Diagnostic evaluation should be prompted in patients with high suspicion (**Figure 1**) (41, 42). Medical history and clinical evaluation, including growth charts in children, are vital in making the initial diagnosis. According to the Endocrine Society guidelines on the diagnostic workup of CS, after excluding exogenous glucocorticoid exposure, cortisol levels should be measured (43). Increased cortisol levels are confirmed with 24-hour urinary free cortisol (UFC) test, late-night salivary cortisol and/or a low-dose dexamethasone suppression test (1mg overnight or 2mg/day over 48 hours). The diagnostic accuracy is not 100% in any those tests and thus, usually multiple tests may be needed in order to establish the diagnosis. In some cases, falsely high UFC (known as pseudo-CS) may be obtained due to severe obesity, depression, pregnancy, alcoholism, chronic exercise, anorexia, anxiety, malnutrition or excessive water intake (>5L/day) (22). Once the presence of endogenous CS has been confirmed, ACTH is used to differentiate between ACTH-dependent and ACTH-independent CS. If ACTH levels are <5pg/mL, then ACTH-independent disease is suggested, while ACTH levels ≥29pg/ml have approximately 70% sensitivity in identifying ACTH-dependent disease in children (35, 43). Following that, high dose dexamethasone suppression test (Liddle test) is used to differentiate between CS due to adrenal causes or CD from ectopic ACTH secretion (44).

When bilateral adrenocortical hyperplasia is suspected then low-dose dexamethasone test (30ug/kg/dose every 6 hours for 8 doses) followed by high-dose dexamethasone test (120 ug/kg/dose every 6 hours for 8 doses) is performed. However, the diagnosis of CS due to PPNAD can be challenging as often has a cyclical or atypical presentation (e.g. normal or near normal 24-hour UFC). What is very useful though is that these patients appear to have a counterintuitive increase in the production of cortisol in response to high-dose dexamethasone through a PKA-mediated mechanism; this results from the overexpression of glucocorticoids receptors on the adrenocortical cells (45). For distinction from pituitary tumors causing CS, diagnostic imaging may be required, including pituitary magnetic resonance imaging (MRI) (46).

### Isolated micronodular adrenocortical disease

iMAD, like PPNAD, can cause CS usually of mild to moderate severity and it can be cyclical (24, 47). It usually occurs earlier than PPNAD and presents during infancy and childhood (2).

Underlying genetic defects include inactivating variants in phosphodiesterases 11A (*PDE11A*) and 8B (*PDE8*) (48–50). *PDE11A* is located on chromosome 2q31.2, encoding a phosphodiesterase that degrades both cAMP and cGMP. It is expressed in several endocrine tissues, including the adrenal cortex (51). Thus far, five inactivating variants in *PDE11A* have been identified, three in patients with iMAD and two in patients with PPNAD (31, 32). Out of these variants, three lead to introduction of premature termination codons, whereas the other two disrupt the catalytic domain of PDE11A. Thus, all variants resulted in compromised cAMP degradation, supporting the notion that aberrantly hyperactive cAMP -dependent signaling has a causative role in the development of adrenocortical tumors. Notably, patients harboring such inactivating variants also had decreased *PDE11A4* expression (52).

Out of the cAMP-specific PDEs, the one with the highest expression in the adrenal gland is PDE8B (53), and missense variants in *PDE8B* have been associated with adrenocortical tumors; the mutant forms of PDE8B have compromised ability to degrade cAMP, resulting in abnormally elevated PKA activity. Specifically, a study of 84 patients with adrenal tumors but no identified variants in *PRKAR1A*, *PDE11A* or *GNAS*, discovered a p.H391A substitution in PDE8B (54). In addition, a p.H305P substitution was found in a two year old girl with CS and iMAD (32). The variant was transmitted to the patient from her father. However, the father’s phenotype was characterized as normal/very mild, echoing findings in female patients who inherited PDE11A variants from unaffected fathers.

### ACTH-independent macronodular adrenal hyperplasia

AIMH/BMAH presenting with CS is more common in older adults and are usually sporadic (47); however, a few familial cases that have been reported appear to be in children (22). It develops progressively over the years with subclinical hypercortisolism (47, 55). In addition, BMAH can rarely occur as part of McCune-Albright syndrome (MAS) where it appears in the infantile period (<6 months old); in a few cases CS spontaneously resolved (24, 56). MAS is due to somatic activating variants in the *GNAS* gene, that encodes the α-subunit of the Gs protein, causing constant non-ACTH-dependent adrenal cortex stimulation (57).

### Congenital adrenal hyperplasias

CAHs is a group of disorders of defective steroidogenesis inherited in an autosomal recessive manner (58). Inactivating variants in genes encoding enzymes that participate in the cortisol synthesis pathway result in a broad spectrum of disease severity; impaired synthesis of cortisol leads to persistently high levels of ACTH *via* negative feedback, overstimulation of the adrenal cortex and eventually adrenal hyperplasia and oversecretion of the precursors of the enzymatic defect. When the enzymatic defects are complete or almost complete overt enzymatic insufficiency ensues and those defects are known as ‘classic’ CAH. The ‘non classic’ CAH (NCCAH; also termed ‘late onset’) tends to be milder as the enzymatic defects are partial and patients retain some cortisol and aldosterone production. In most cases, patients with NCCAH harbor either two non-classic alleles or one non-classic and one classic, meaning that when two individuals with NCCAH have kids their risk of having a child with classic CAH is approximately 1.5-2.5% (59, 60).

The prevalence of the classic forms is 1:16,000 while for the non-classic forms is approximately 1:2,000 in the Caucasian population in the United States with a higher frequency in Hispanics, Eskimos, people of Mediterranean or Middle-Eastern descent and Ashkenazi Jews (61–65).

#### 21-hydroxylase deficiency

21OHD (OMIM#201910, *CYP21A2*) accounts for approximately 95% of CAH cases (61, 66, 67). The enzyme 21OH catalyzes the conversion of 17-hydroxyprogesterone to 11-deoxycortisol in the ZF and progesterone to 11-deoxycorticosterone (DOC) in ZG. *CYP21A2* is located on chromosome 6p21.3, within the human leukocyte antigen (HLA) locus. About 30kb apart, the same locus harbors a non-functional highly homologous pseudogene (*CYP21A1P*). Because of that, recombination frequently occurs in the region; these recombination events are responsible for most of the *CYP21A2* inactivating variants in patients.

21OHD presents with a wide spectrum of phenotypes. Increased ACTH secretion due to inadequate production of cortisol leads to accumulation of 17-hydroxyprogesterone and progesterone. As a result, female infants with classic 21OH deficiency are exposed to excess androgens *in utero*; at birth, they present with virilization of the genitalia, including rugated or partially fused labia majora, genital hyperpigmentation, enlarged clitoris and a vagina that opens into a common urogenital sinus -like in males- while the ovaries, fallopian tubes and the uterus are normal (61). Males have minimal findings, including genital hyperpigmentation and macrogenitosomia or no physical findings of the disease (61, 68).

Linear growth is also affected as patients are at risk for centrally mediated precocious puberty due to prolonged exposure to high levels of androgens. Data from 18 centers demonstrated that their adult height is on average 1.4SD below the mean compared to the general population (69). Early diagnosis and treatment compliance are vital as both overtreatment and undertreatment increase the risk for short stature, either due to excess glucocorticoid-induced inhibition of the growth axis or due to premature epiphyseal closure (70, 71).

Approximately 75% of classic CAHs represent life-threatening salt-wasting forms and they are a result of large gene conversions, nonsense or frameshift mutations or complete deletions leading to complete absence of 21OH activity. Production of mineralocorticoids and glucocorticoids is ceased resulting in life-threatening adrenal crises in the first two weeks of life (72). When CAH is suspected, then aldosterone, serum electrolytes and plasma renin should be measured. The expected abnormalities are low aldosterone levels, hyperkalemia and elevated renin; however, because renin levels are age-specific appropriate references should be used (73). On the other hand, the NCCAH forms are more often associated with missense variants and they retain some enzymatic activity and thus are able to maintain a relatively normal amount of aldosterone and cortisol at the expense of mild to moderate excess of sex hormone precursors salt balance (72); they present with premature pubarche during childhood; however cases have been described as early as six months old (74). Females most commonly present during adolescence with hirsutism, acne and menstrual abnormalities or infertility, findings similar to polycystic ovarian syndrome (PCOS) while males often remain undiagnosed until they undergo pre-conception genetic screening or after having an affected offspring (74).

#### CAH-tenascin-X syndrome

Some of the deletions responsible for CAH also lead to Ehlers-Danlos syndrome (hypermobility type). This occurs when the deletion encompasses both *CYP21A2* and *TNXB* (which encodes tenascin-X; a protein of the extracellular matrix). In such cases, the resulting contiguous gene deletion syndrome is called CAH-X syndrome. Overall, CAH-X is estimated to have a prevalence of 9% among CAH patients.

More specifically, there exist 3 subtypes of CAH-X: a) CAH-X CH-1, associated with deletion of exon 35 of TNXB; b) CAH-X CH-2, caused by the c.12174 C>G (p.Cys4058Trp) variant in *TNXB*; and c) CAH-X CH-3, which is caused by a cluster of 3 variants. The mechanism of two latter subtypes involves dominant negative effects.

#### CAH due to other, less common defects in steroidogenic enzymes

##### 17α-hydroxylase deficiency

17α-hydroxylase (CYP17A1) catalyzes two reactions, the 17-hydroxylase and the 17,20-lyase reaction. Pathogenic variants in *CYP17A1* (including splice site alterations, point mutations, small insertions/deletions, and in rare cases, large deletions) result in impairment of both reactions.

Complete 17OHD (OMIM#202110) leads to disrupted steroidogenesis in both the adrenals and the gonads, as it is expressed in both, leading to puberty failure. In addition, both 46,XX and 46,XY will present with female external genitalia due to absent testosterone and dihydrotestosterone; however, 46,XY individuals, due to preservation of anti-Mullerian hormone from the testes do not have an internal Mullerian structure (75). In this form of CAH, in contrast with the others, there is some glucocorticoid production from corticosterone and thus adrenal crisis is very rare. Over the years, accumulation of DOC causes hypokalemia and hypertension. In general, the typical presentation of 17OHD is a girl in adolescent with no secondary sexual characteristics and low-renin hypertension (76, 77). Non-classic forms of 17OHDy have not been well described (75).

##### 11β-hydroxylase deficiency

About 0.2-8% of all CAH cases are due to 11βOHD (OMIM#202010) (72). 11βOH encoded by *CYP11B1*, catalyzes the conversion of 11-deoxycortisol and 11-deoxycorticosterone (DOC) to cortisol and corticosterone, respectively. Impaired cortisol and corticosterone production leads to overproduction of the precursors that are later converted to androgens causing virilization in females (78). In addition, patients present with mild to moderate hypertension. Although deoxycorticosterone is not as a potent mineralocorticoid as aldosterone, when it accumulates it leads to retention of salt and hypokalemic hypertension (79).

##### 3β-hydroxysteroid dehydrogenase type 2 deficiency (also known as HSDB)

This form of CAH is very rare accounting for <0.5% of the cases (OMIM#201810) (80). 3β-hydroxysteroid dehydrogenase exists in two isoforms; 3βHSD1, encoded by *HSD3B1* (the homologous type I gene) and is expressed in the placenta and peripheral tissue (liver, skin, brain) and 3βHSD2, encoded by *HSD3B2* and is found in the adrenals and gonads (72). Inactivating variants in *HSD3B2* impair steroidogenesis in both gonads and adrenals, leading to deficiency of glucocorticoids and mineralocorticoids as well as overproduction of dehydroepiandrosterone (DHEA). Subsequently, DHEA is converted to testosterone by 3βHSD1 and thus both 46,XX and 46,XY present with ambiguous genitalia at birth (81). In rare cases, 46,XX can present with virilization, including incomplete fusion of the labia, enlarged clitoris and hyperpigmentation of the genital region due to the increased testosterone (72).

##### Steroidogenic acute regulatory protein deficiency

StAR is a mitochondrial protein that regulates cholesterol influx between the outer and inner mitochondrial membrane, a critical and first step of steroid hormone synthesis (82). Inactivating variants in *STAR* gene cause Lipoid Congenital Adrenal Hyperplasia (LCAH, the most severe form of CAH, OMIM#201710) (83). The adrenals are filled with lipid globules derived from cholesterol and thus the name of the disease. Patients with LCAH have impaired production of mineralocorticoids, glucocorticoids and sex steroids. LCAH appears to be more common in the Korean, Japanese and Palestinian Arab populations (83–92), while certain variants have much higher frequency in specific ethnic groups, including p.Q258X variant in Japanese and Koreans (86, 89) as well as p.R182L variant in Palestinian Arabs (83). Both 46,XX and 46,XY present with female or ambiguous genitalia and adrenal crises during the neonatal period. Hyperpigmentation is also frequent due to the increased levels of ACTH. However, milder forms of the disease have been described in the literature that presented with late-onset adrenal insufficiency and male genitalia (93).

#### Diagnosis

21OHD can be diagnosed *via* the measurement of 17-hydroxyprogesterone; this measurement does not need necessarily to be performed after ACTH stimulation. Hormonal testing is also used to diagnose the rarer forms of CAH. Because carriers cannot be reliably identified using biochemical methods, genetic testing is necessary to detect heterozygous individuals, who can then be offered genetic counseling. Specifically for the salt-wasting form of CAH, the United States and more than 35 countries around the world have implemented newborn screening programs for early detection and treatment (94).

## Conclusion

Adrenocortical hyperplasias are rare in children. Advances over the past two decades have led to a better understanding of their molecular background, including the characterization of the cAMP/PKA signaling pathway as the main component of their pathogenesis. CAH are a group of rare diseases with genetic and clinical heterogeneity and can be life-threatening in their most severe form. The molecular analysis of CAH is useful in confirming the diagnosis and provides a powerful tool in genetic counseling.

## Author contributions

GP: Writing-original draft and editing; CAS.: Conseptualization, supervision, writing, review and editing.

## Funding

This study was supported by the Intramural Research Program, Eunice Kennedy Shriver National Institute of Child Health & Human Development (NICHD), Bethesda, MD, United States. This work was in part supported by the research project Z01-HD008920 (Principal Investigator: Constantine A Stratakis) of the Intramural Research Program of the Eunice Kennedy Shriver National Institute of Child Health & Human Development (NICHD), National Institutes of Health (NIH), Bethesda, MD, USA. Stratakis is currently also funded by IMBB, FORTH intramural funds.

## Conflict of interest

Author CS holds patents on the *PRKAR1A*, *PDE11A* and *GPR101* genes and/or their function and has received research funding from Pfizer Inc. on the genetics and treatment of abnormalities of growth hormone secretion. CAS is receiving compensation by ELPEN, Inc. Neither Pfizer, Inc nor ELPEN, Inc had any role in the study design, data collection and analysis, decision to publish, or preparation of the manuscript.

The remaining author declares that the research was conducted in the absence of any commercial or financial relationships that could be construed as a potential conflict of interest.

## Publisher’s note

All claims expressed in this article are solely those of the authors and do not necessarily represent those of their affiliated organizations, or those of the publisher, the editors and the reviewers. Any product that may be evaluated in this article, or claim that may be made by its manufacturer, is not guaranteed or endorsed by the publisher.
